# Supplementary material for: Chromosome-encoded IpaH ubiquitin ligases indicate non-human enteroinvasive Escherichia
Source: Sci Rep. 2022 Apr 27;12:6868. doi: 10.1038/s41598-022-10827-3 (PMC9046306; doi:10.1038/s41598-022-10827-3)
Supplement: Supplementary file 1 — Supplementary Figures. [file 41598_2022_10827_MOESM1_ESM.pdf]

Supplementary Figures

Chromosome-encoded IpaH ubiquitin ligases indicate non-human enteroinvasive Escherichia

Authors: Natalia O. Dranenko[1], Maria N. Tutukina[1,2,3], Mikhail S. Gelfand[1,2], Fyodor A. Kondrashov[4], Olga O. Bochkareva[4#]

Affiliations:

[1] A.A. Kharkevich Institute for Information Transmission Problems, Moscow, Russia

[2] Skolkovo Institute of Science and Technology, Moscow, Russia

[3] Institute of Cell Biophysics, Russian Academy of Sciences, FRC PSCBR RAS, Moscow Region, Pushchino, Russia

[4] Institute of Science and Technology (IST Austria), Klosterneuburg, Austria

[#] Correspondence: *olga.bochkareva@ist.ac.at*

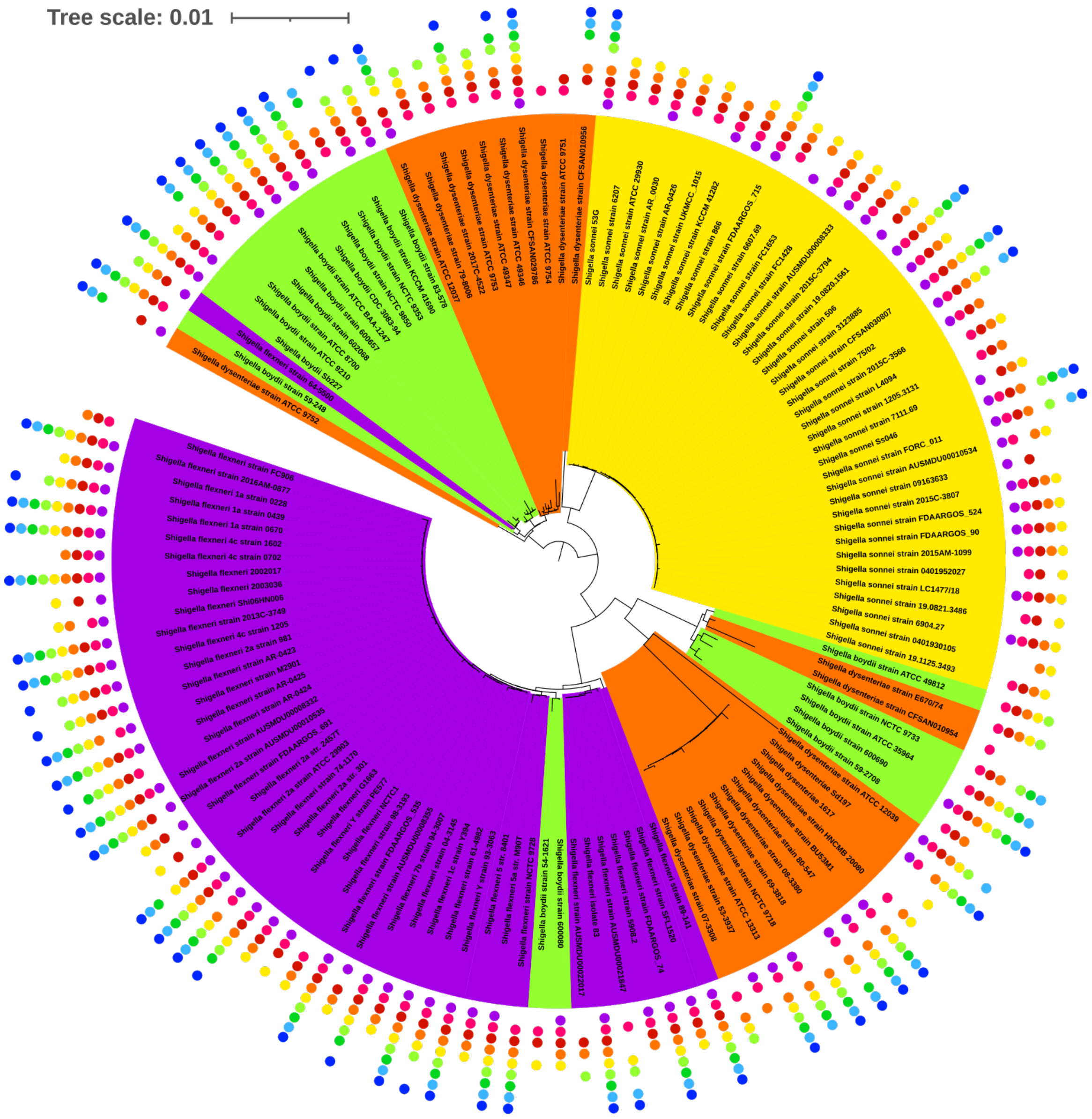

Supplementary Figure S1*. Phylogenetic tree of *Shigella* spp.** colored by species name: violet - *S. flexneri*, green - *S. boydii*, orange - *S. dysenteriae*, yellow - *S. sonnei*. Other notation as in ***Figure 3.

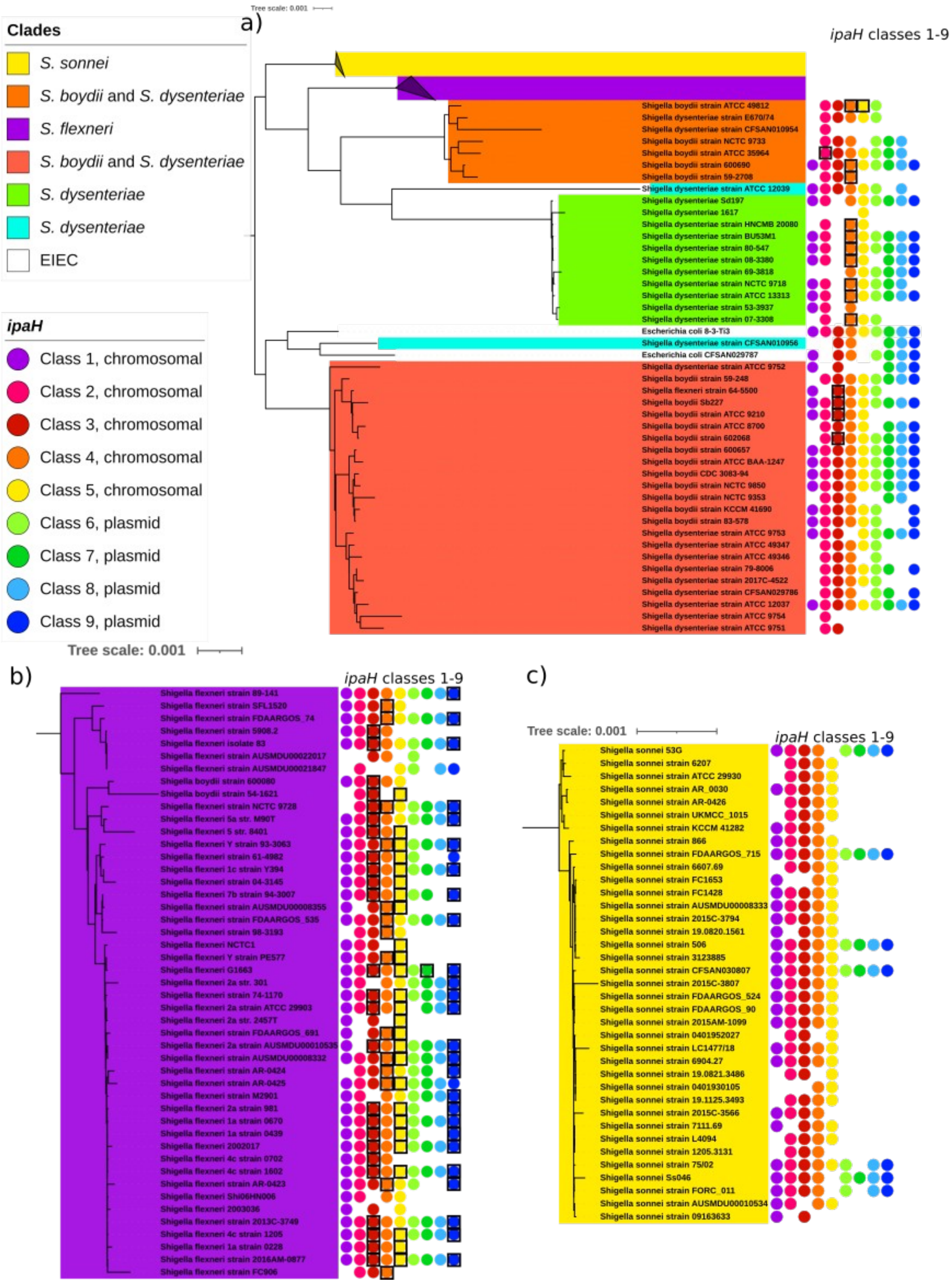

Supplementary Figure S2. **Abundance of the *ipaH* family members in *Shigella* clades.** The notation as in ***Figure 3***. Black frames indicate duplications. Pannels (a), (b), and (c) reflect three major tree clades.

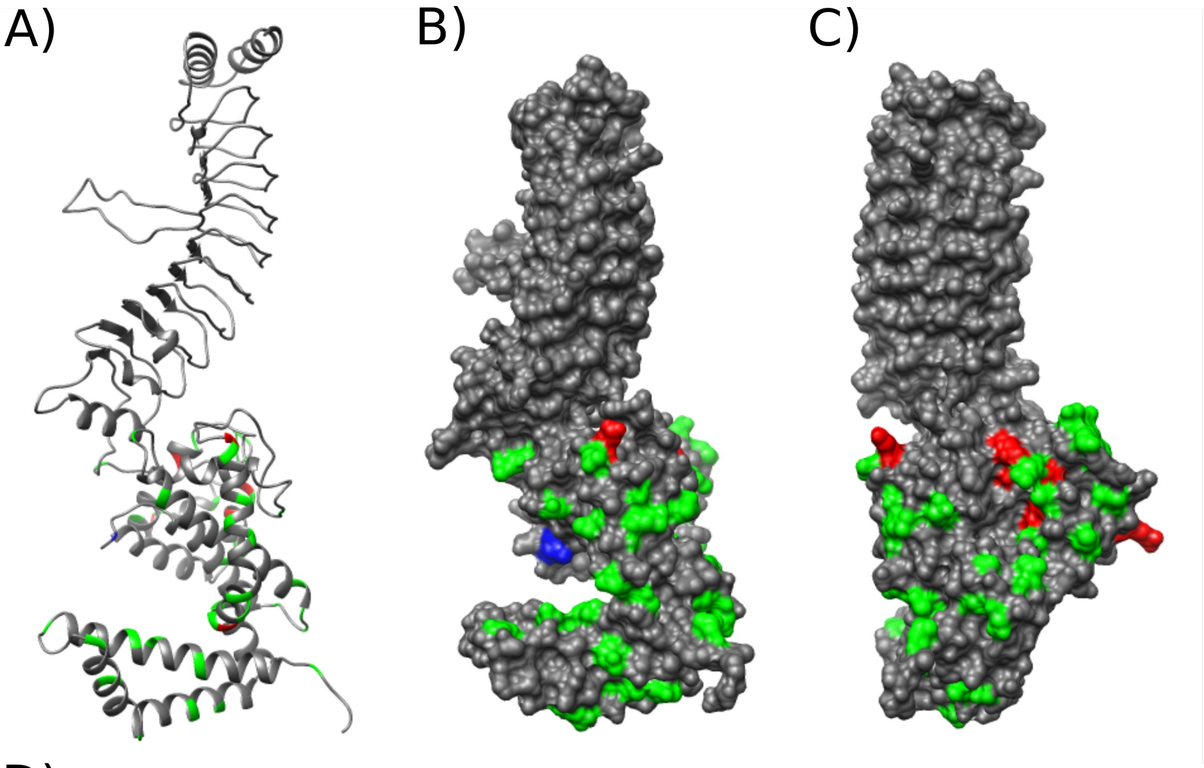

D)

```
ShigellaCdomain    GPQIYFSMSDGQQNTLHRPLADAVTAWFPENKQSDVSQIWHAFEHEEHANTFSAFLDRLS
Non-humanCdomain1 GPQIHFSMSDGQQHTSVRPLPEAVAAWFPQNKQSDVSQIWLAFEREEHANTFSAFLDRLA
Non-humanCdomain2 GPQIHFSMSDGQQHTPVRSLPEAVAAWFPESLRSEVSQRWAAFTDEENAATFSAFLDRLA

ShigellaCdomain    DTVSARNTSGFREQVAAWLEKLSTSAELRQQSFAVAADATESCEDRVALTWNNLRKTLLV
Non-humanCdomain1 DTVSARNAQGFRQQVSAFLEKLSTSAELRQQSFAVAADATESCEDRVALTWNNLQKSLLV
Non-humanCdomain2 DTVTARNAPGFAQQVSEFLEKLSMSEALRQQCFAVAADATRSCEDRVALTWNNLQKTYRV

ShigellaCdomain    HQASEGLFDNDTGALLSLGREMFRLEILEDIARDKVRTLHFVDEIEVYLAFQTMLAEKLQ
Non-humanCdomain1 HQASEGLFDNDTGALLSLGREMFRLEILEDIARDKVRTLRFVDEIEVYLAFQTMLAEKLQ
Non-humanCdomain2 HQASEGEFDSDLTGLLSLGREMYRLEVLEEIAREKVRTLHFVDEIEVSLAFQTMLAEKLE

ShigellaCdomain    LSTAVKEMRFYGVSGVTANDLRTAEAMVRSREENEFTDWFSLWGPWHAVLKRTEADRWAQ
Non-humanCdomain1 LSTAVKEMRFYGVSGVTENDLRTAEAMVRSREENEFTDWFALWGPWHAVLKRTEADRWAQ
Non-humanCdomain2 LSTAVREMRFYGVSGVTEDDLSSALVRVLSREEREFAEWFARWSPWHAVLKRTEAERWAR

ShigellaCdomain    AEEQKYEMLENEYPQRVADRLKASGLSGDADAEREAGAQVMRETEQQIYRQLTDEVLALR
Non-humanCdomain1 AEEQKYEMLENEYPQRVADRLKASGLSGDADAEREAGAQVMRETEQQIYRQLTDEVLALR
Non-humanCdomain2 AEEKKYEMLERKYPQRLAERLSALDLSGDGDAEREAGVRVMEEIETEIYRQLTEEVSGER

ShigellaCdomain    RLPENGSRLHHS
Non-humanCdomain1 RLPENGSQLHHS
Non-humanCdomain2 RLAENRAR----
```

Supplementary Figure S3. **Homology modeling of IpaH from *Escherichia marmotae.***

The predicted protein structure of *Escherichia marmotae* IpaH class 1 **(A)** as a ribbon representation; **(B)** with surface of the protein as seen from the binding site side; **(C)** with surface of the protein from the side opposite to the binding site. **(D)** Sequence alignment of three variants of the C-terminal domains from *Shigella* spp. and *Escherichia marmotae*. The active site is marked in blue. The

positions strongly shared between two domains from *E. marmotae* but diverse in *Shigella* are marked in red; the positions strongly shared between *Shigella* and *Shigella*-type domain from *E. marmotae* are marked in green.
